# Associations of Rheumatoid Arthritis Disease Activity With Frailty Over Five Years of Follow‐up

**DOI:** 10.1002/acr.80056

**Published:** 2026-05-05

**Authors:** Courtney N. Loecker, Yangyuna Yang, Punyasha Roul, Aaron Baraff, Myra S. Schmaderer, Lani Zimmerman, Kaleb Michaud, Namrata Singh, Grant W. Cannon, Gary Kunkel, Ted R. Mikuls, Ariela R. Orkaby, Joshua F. Baker, Bryant R. England, Katherine D. Wysham

**Affiliations:** ^1^ College of Nursing, University of Nebraska Medical Center Lincoln Nebraska; ^2^ VA Nebraska‐Western Iowa Health Care System Omaha Nebraska; ^3^ Division of Rheumatology & Immunology, Department of Internal Medicine University of Nebraska Medical Center Omaha Nebraska; ^4^ Arthritis Section, VA Puget Sound Health Care System Seattle Washington; ^5^ Department of Statistics University of Washington Seattle Washington; ^6^ FORWARD, The National Databank for Rheumatic Diseases Wichita Kansas; ^7^ Division of Rheumatology, Department of Medicine University of Washington Seattle Washington; ^8^ VA Salt Lake City Health Care System Salt Lake City Utah; ^9^ VA Boston Healthcare System & Brigham and Women's Hospital, Harvard Medical School Boston Massachusetts; ^10^ New England VA Boston Healthcare System Geriatric Research Education and Clinical Center Jamaica Plain Massachusetts; ^11^ University of Pennsylvania & Corporal Michael J. Crescenz VA Medical Center Philadelphia Pennsylvania; ^12^ VA Puget Sound Geriatric Research Education and Clinical Center Seattle Washington

## Abstract

**Objective:**

To evaluate whether rheumatoid arthritis (RA) disease activity is associated with frailty both in cross‐section and longitudinally.

**Methods:**

Participants within the Veterans Affairs Rheumatoid Arthritis (VARA) registry enrolled from 2003 to 2022 were included. The exposure was RA disease activity measured by the Disease Activity Score in 28 joints (DAS28) with erythrocyte sedimentation rate or C‐reactive protein. DAS28 was collected at VARA registry enrollment, and the mean DAS28 score was calculated annually and categorized by level of disease activity. The outcome was frailty measured using the Veterans Affairs Frailty Index (VAFI), which was calculated annually from diagnostic and procedural codes. The relationship of DAS28 with VAFI score was examined at baseline and over five years of follow‐up. Multivariable linear regression was used for cross‐sectional analysis and generalized estimating equations evaluated the associations longitudinally.

**Results:**

Of the 2,800 veterans, 89% were male, 76% were White, the mean age was 64 ± SD 11 years, average RA disease duration was 12 ± SD 11 years, and 28% were frail. A higher DAS28 score was associated with an increasing VAFI score at enrollment (adjusted β 0.86, 95% confidence interval [CI] 0.63–1.08, *P* < 0.001, N = 2,800). RA disease activity scores were independently associated with frailty scores over five years of follow‐up (adjusted β 0.53, 95% CI 0.26–0.80, *P* < 0.001, n = 2,786).

**Conclusion:**

Higher RA disease activity was associated with increased frailty burden over time. Future studies should assess whether achieving low disease activity or remission can impact frailty burden over time.

## INTRODUCTION

Frailty has increasingly been recognized as a salient concept in people with rheumatoid arthritis (RA). Frailty is an age‐associated vulnerability to adverse health outcomes that occurs prematurely and has twice the prevalence in adults with RA when compared to older cohorts in the general population.^1^, ^2^, ^3^ Frailty in RA is associated with an increased risk for several poor long‐term outcomes including greater physical impairment,^4^ fractures,^5^ unemployment,^6^ hospitalizations,^5^, ^7^, ^8^ and death.^7^, ^8^ RA disease activity is crucial to consider when assessing frailty because poorly controlled RA can manifest as joint inflammation and pain, impaired mobility, weakness, and fatigue, making people more susceptible to frailty.^9^, ^10^

**SIGNIFICANCE & INNOVATIONS**

- This study is among the first to evaluate the association between rheumatoid arthritis (RA) disease activity and frailty using repeat measures over long‐term follow‐up in people with established RA.
- We demonstrated that individuals with higher RA disease activity were more likely to experience greater frailty burden over five years of follow‐up.
- Lowering RA disease activity in adults with RA could help prevent or mitigate frailty and promote healthy aging in this population.
- Future studies are needed to determine whether achieving remission or low disease activity can modify frailty burden in RA.

Most studies examining RA disease activity and frailty have been cross‐sectional using varied frailty and disease activity instruments, leaving speculation about whether disease activity impacts frailty burden over time.^1^, ^4^, ^11^, ^12^ The Frailty Phenotype and Frailty Index (FI) are measures most frequently used to assess frailty in adults with RA.^13^ The Frailty Phenotype originated in the Cardiovascular Health Study identifying frailty as the presence of three of five criteria: unintentional weight loss, weakness (weak grip strength), exhaustion, slow walking speed, and low physical activity.^14^ This method of frailty measurement has been criticized for its unidimensional focus on physical frailty and impracticality in clinical settings because of the time and equipment required for implementation.^15^ The FI postulates frailty as an accumulation of health deficits across multiple domains that can be derived from existing clinical or administrative records with a higher FI score indicating greater frailty.^3^, ^13^ Early improvement of FI scores has been described in patients with newly diagnosed RA owing to treatment initiation.^7^ Frailty burden and RA disease activity were higher after two years in frail versus nonfrail people despite initial improvement.^7^ In addition to the brief follow‐up duration, morbidities were carried forward in the repeat FI scores, which may have underestimated true frailty burden, and the statistical significance of the relationship between RA disease activity and frailty was not described.^7^ Although frailty has been linked to RA disease activity and poor outcomes, the longitudinal relationship between RA disease activity and frailty is incompletely understood.^1^, ^4^

To address these knowledge gaps, the aim of this study was to evaluate the relationship more precisely between RA disease activity and frailty using a clinically practical frailty instrument while leveraging repeat measures and controlling for important confounders. We hypothesized that participants with higher RA disease activity would demonstrate higher frailty at Veterans Affairs Rheumatoid Arthritis (VARA) registry enrollment and over time. Characterizing the association between RA disease activity and frailty over time has potential to inform the development of targeted interventions to help mitigate frailty burden and improve outcomes.

## MATERIALS AND METHODS

### Study design and data sources

Cross‐sectional and longitudinal study designs were performed, using data from the VARA registry, a prospective, multicenter cohort study initiated in 2003 with 17 participating rheumatology clinics from VA Medical Centers across the United States.^16^ VARA includes a cohort of veterans with prevalent RA, the RA diagnosis fulfilling 1987 or 2010 American College of Rheumatology (ACR) criteria, who receive rheumatology care at a VA Medical Center.^17^, ^18^ Patient sociodemographic and RA disease history were collected both by patient report and by rheumatologist investigators at the time of VARA enrollment. ACR core RA disease activity measures including 28 tender and 28 swollen joint counts, patient global assessment (PtGA) and provider global assessment (PrGA), and biomarkers were collected at enrollment and during routine clinic visits. VA administrative data (ie, *International Classification of Disease* codes, procedure codes) and pharmacy data within the VA Corporate Data Warehouse were linked to the VARA registry. Data were housed in the central repository and accessed securely by VARA researchers and collaborators in the VA Informatics and Computing Infrastructure (VINCI).^16^ Data from 2003 to 2022 were included in the study. We required participants to have a VA Frailty Index (VAFI) score at enrollment and one or more RA disease activity measure(s) in the subsequent five years following enrollment. Veterans missing VAFI scores at VARA enrollment (n = 14) were excluded.

### Ethical approval

All participants provided written informed consent, and each study site received local institutional review board approval.

### Disease activity measure

The independent variable was RA disease activity measured using the Disease Activity Score in 28 joints (DAS28) ranging from 0 to 9.4 with the following disease activity levels: remission <2.6, low disease activity 2.6 to <3.2, moderate disease activity 3.2 to <5.1, and high disease activity ≤5.1.^19^ The DAS28 with erythrocyte sedimentation rate (ESR) was used preferentially, but if only a C‐reactive protein (CRP) was available, the DAS28 with CRP was used. The DAS28 score(s) were collected at VARA registry enrollment and routine visits as dictated by usual care.^16^ The nearest nonmissing DAS28 value before or after the index visit was used if there was no DAS28 value at enrollment. Because individuals may have more than one DAS28 assessment over the course of one year, mean DAS28 values were calculated over the subsequent years annually for up to five years after enrollment for the longitudinal analyses. In addition to the composite DAS28, the individual components of RA disease activity were collected at enrollment from the VARA registry including swollen joint count (SJC; 0–28), tender joint count (TJC; 0–28), PtGA (0–100), PrGA (0–100), and ESR (mm/h) or CRP (mg/dL). Similar to the composite DAS28 score, when the individual component value was missing at enrollment, the nearest nonmissing value before or after the index visit was used. In the regression models, the components PtGA, PrGA, and ESR were divided by 10 to allow for easier interpretation. Throughout this manuscript, baseline RA disease activity refers to disease activity at VARA registry enrollment.

### Study outcomes

The dependent variable was frailty measured using the validated 31‐item VAFI score, which used diagnostic and procedure codes linked to health deficits encompassing morbidity, functional status, cognition and mood, sensory impairment, and other geriatric syndromes of the prior three years.^20^, ^21^, ^22^, ^23^ The composite VAFI score used in our analyses was determined by calculating the number of deficits present divided by the 31 possible deficits, ranging from 0 to 1 with scores >0.20 classifying frail.^20^ VAFI scores were housed in VARA, which used data from national VA databases to extract VA administrative codes to calculate the VAFI using a three‐year lookback of inpatient and outpatient diagnoses and procedures (Supplement 1). The VAFI was calculated at registry enrollment and annually thereafter until death or end of the study period. In the longitudinal models, a three‐year lag between the disease activity and VAFI was incorporated to avoid reverse causation, ensuring VAFI health deficits were obtained after the disease activity measure (Figure 1).

**Figure 1:**
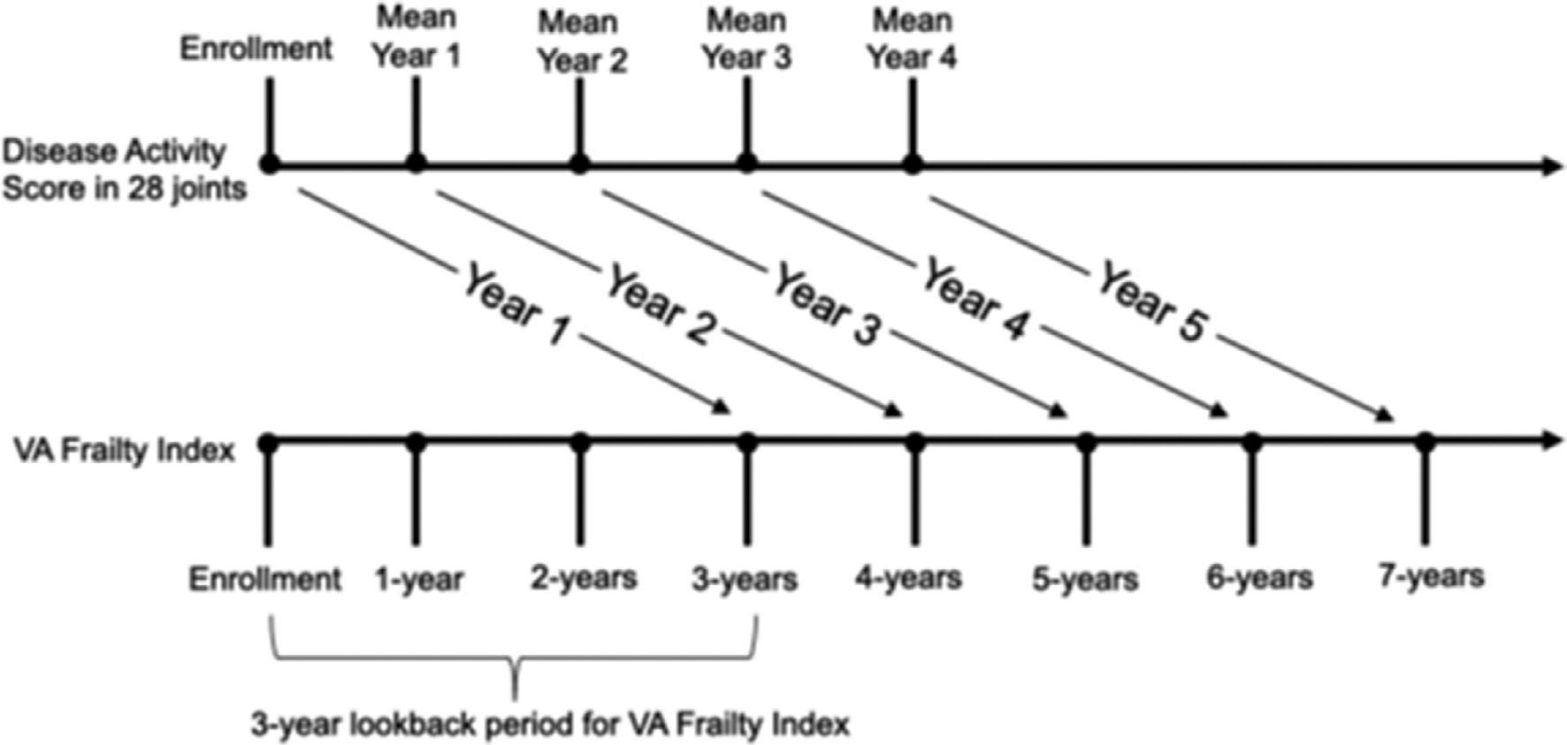
Schematic representation of the model used to analyze the longitudinal association between rheumatoid arthritis disease activity and frailty. To ensure temporality, each mean disease activity measurement was aligned with the VA Frailty Index outcome occurring after a 3‐year lookback period, resulting in a 3‐year lag between exposure and outcome. VA, Veterans Affairs.

### Other measures

Other measures were age category (<60 years, 60–69 years, 70–79 years, ≥80 years); sex; self‐reported race categorized as Black, White, or other to include American Indian or Alaska Native, Asian, multiracial, and Native Hawaiian or other Pacific Islander; education (≤12 years, >12 years); RA disease duration (years); body mass index (BMI; kg/m^2^); smoking status (never, current, former); Multidimensional Health Assessment Questionnaire (MDHAQ); Clinical Disease Activity Index (CDAI); and anti–cyclic citrullinated peptide (anti‐CCP) positivity based on standardized assays.^1^, ^4^ Age was analyzed as a categorical variable because it does not have a linear relationship with the outcome of frailty.^23^ The CDAI (range 0–76) was scored from the sum of the SJC (0–28), TJC (0–28), PtGA (0–10), and PrGA (0–10) scores. Disease activity levels were interpreted as follows: remission ≤2.8, low disease activity >2.8 to 10, moderate disease activity >10 to 22, and high disease activity >22.^24^ Covariates were selected a priori based on existing literature and content expertise that may confound the relationship between RA disease activity and frailty. Covariates were fixed at baseline values and collected from the VARA registry.

### Statistical analyses

Descriptive statistics were used to assess baseline characteristics overall and for frail and nonfrail participants. Frequencies and percentages were reported for categorical variables. Mean and SDs were reported for continuous variables.

#### Cross‐sectional analyses

Multivariable linear regression was used to evaluate the association between the composite (continuous) DAS28 score and VAFI score at baseline before and after adjusting for age, sex, race, education level, BMI, smoking status, RA disease duration, and anti‐CCP seropositivity. We repeated the primary cross‐sectional analysis substituting disease activity (DAS28) category for continuous disease activity score.

A secondary exploratory analysis evaluated the relationship between RA disease activity component(s) and frailty. The primary analyses were repeated substituting the composite DAS28 with the individual components of RA disease activity: TJC, SJC, PtGA, PrGA, ESR, and CRP. Components were evaluated in separate linear regression models due to concern for multicollinearity.

In a sensitivity analysis, the primary analyses were repeated adding RA medications to the original model to explore whether the model was substantially affected by medication use. These models additionally included prednisone, conventional synthetic disease‐modifying antirheumatic drug (csDMARD; methotrexate, hydroxychloroquine, sulfasalazine), and biologic and targeted synthetic DMARD (b/tsDMARD; abatacept, adalimumab, etanercept, infliximab, rituximab, tocilizumab, tofacitinib) use. In an additional sensitivity analysis, the CDAI was substituted for the DAS28 (composite score and then categories of RA disease activity) in the original models to assess for robustness of findings when using another RA disease activity measure.

#### Longitudinal analyses

Descriptive statistics were used to assess the distribution of the VAFI (mean, SD, range) and the proportion of frail and nonfrail participants annually over up to five years of observation. The association of DAS28 score with frailty score was evaluated at enrollment and over up to five years of observation using generalized estimation equations (GEE) to account for the correlation of these measures within an individual participant over time (Figure 1).^25^ GEE models with a Gaussian distribution, identity link, and robust estimates were adjusted for baseline covariates (age, sex, race, education level, BMI, smoking status, RA disease duration, and anti‐CCP antibody positivity).^26^ We tested for an interaction between RA disease activity and follow‐up duration (year) with a prespecified significance level of 0.20 in the unadjusted and fully adjusted models to explore whether the association between disease activity and frailty differed across years of follow‐up.

In a sensitivity analysis, the fully adjusted model was repeated with the VAFI using a shortened one‐year lookback of procedural and diagnostic codes and lagged by one‐year after the DAS28 score to assess for robustness of findings (Figure 2).^21^ Similar to the primary model, the interaction term was tested between RA disease activity and follow‐up duration (year) with a significance level of 0.20.

**Figure 2:**
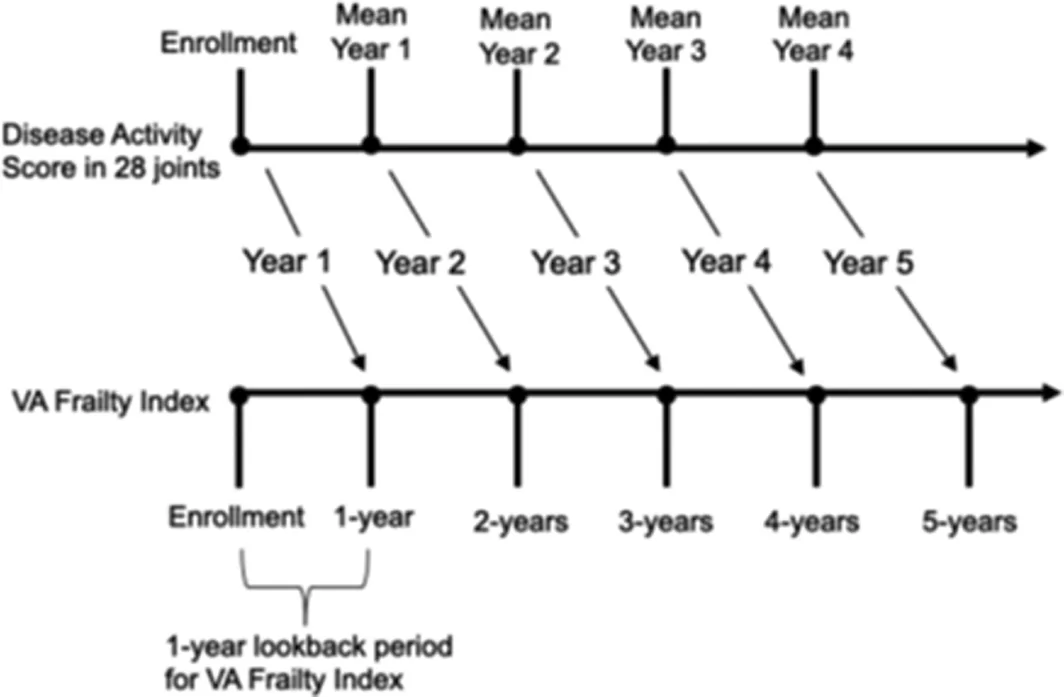
Schematic representation of the model used to analyze the longitudinal association between rheumatoid arthritis disease activity and frailty, using an alternative VA Frailty Index that accumulated codes over a 1‐year lookback period. To ensure temporality using the 1‐year lookback period, each mean disease activity measurement was aligned with the VA Frailty Index outcome occurring after a 1‐year lookback period, resulting in a 1‐year lag between exposure and outcome. VA, Veterans Affairs.

Multiple imputation chained equations technique with 10 iterations was used to handle missing data in regression models.^27^ The level of statistical significance was set at *P* < 0.05. Beta coefficients were multiplied by 100 for readability. All analyses were completed using Stata version 18 within the VINCI platform.^28^

No data other than what are included in this article or supplementary material will be shared. The VA regulatory rules prohibit sharing of data.

## RESULTS

### Participant characteristics

Of the 2,800 veterans with RA studied, the majority were male (n = 2,480, 89%) and White (n = 2,113, 76%), with an average age of 64.1 ± SD 11.0 years. Most had a smoking history (n = 2,156, 79%), and average RA disease duration was 11.5 ± SD 11.4 years. RA disease activity measured by the DAS28 showed 660 veterans (24%) were in remission, 436 (16%) had low disease activity, 1,085 (40%) had moderate disease activity, and 528 (20%) had high disease activity at enrollment. According to the VAFI, 785 veterans (28%) with RA were frail, including 117 (15%) who were younger than 60 years old. Frail participants were older (mean 68.6 ± SD 9.5 years vs mean 62.3 ± SD 11.1 years) with greater functional impairment (mean MDHAQ 1.0 ± SD 0.6 vs 0.8 ± SD 0.6) compared to nonfrail participants (Table 1). Missingness of data is shown in Table 1. All variables had <5% missingness except education level (11%), PtGA (11%), and PrGA (37%).

**Table 1:** Participant demographics and clinical factors by frailty status at VA Rheumatoid Arthritis registry enrollment*

	Nonfrail	Frail	Overall
	n = 2,015 (72%)	n = 785 (28%)	N = 2,800 (100%)
VA Frailty Index (0–1), mean (SD)	0.12 (0.05)	0.30 (0.07)	0.17 (0.10)
Age, mean (SD), y	62.3 (11.1)	68.6 (9.5)	64.1 (11.0)
< 60, n (%)	734 (36)	117 (15)	851 (30)
60–69, n (%)	774 (38)	306 (39)	1,080 (39)
70 to 79, n (%)	412 (20)	258 (33)	670 (24)
≥ 80, n (%)	95 (5)	104 (13)	199 (7)
Male, n (%)	1,780 (88)	700 (90)	2,480 (89)
Race, n (%)			
White	1,511 (76)	602 (78)	2,113 (76)
Black	298 (15)	129 (17)	427 (15)
Other^a^	190 (10)	44 (6)	234 (8)
Education, n (%)			
≤12 y	900 (50)	362 (53)	1,262 (51)
Body mass index, mean (SD), kg/m^2^	28.6 (5.5)	28.8 (6.0)	28.6 (5.6)
Underweight (<18.5), n (%)	23 (1)	11 (2)	34 (1)
Normal weight (18.5–24.9), n (%)	471 (24)	206 (27)	677 (25)
Pre‐obesity (25–29.9), n (%)	786 (40)	248 (33)	1,034 (38)
Obesity class I (30–34.9), n (%)	452 (23)	164 (22)	616 (23)
Obesity class II (35–39.9), n (%)	150 (8)	91 (12)	241 (9)
Obesity class III (≥ 40), n (%)	76 (4)	36 (5)	112 (4)
Smoking status, n (%)			
Never	431 (22)	143 (19)	574 (21)
Current	544 (28)	144 (19)	688 (25)
Former	990 (50)	478 (63)	1,468 (54)
RA disease characteristics			
Disease duration, mean (SD), y	11.1 (11.0)	12.5 (12.3)	11.5 (11.4)
Anti‐CCP seropositivity, n (%)	1,535 (79)	545 (72)	2,080 (77)
MDHAQ, mean (SD)	0.8 (0.6)	1.0 (0.6)	0.9 (0.6)
RA disease activity measures			
DAS28, mean (SD)	3.7 (1.5)	4.0 (1.5)	3.8 (1.5)
Remission, n (%)	531 (27)	129 (17)	660 (24)
Low, n (%)	313 (16)	123 (17)	436 (16)
Moderate, n (%)	768 (39)	317 (42)	1,085 (40)
High, n (%)	350 (18)	178 (24)	528 (20)
CDAI, mean (SD)	14.4 (12.4)	16.3 (13.2)	14.9 (12.7)
RA disease activity components, mean (SD)			
Tender joint count (0–28)	4.6 (6.5)	4.9 (6.7)	4.7 (6.5)
Swollen joint count (0–28)	3.8 (5.3)	3.6 (5.0)	3.7 (5.2)
Patient global (0–100)	37.6 (25.4)	44.0 (26.0)	39.4 (25.7)
Provider global (0–100)	31.8 (22.6)	35.2 (23.7)	32.8 (23.0)
ESR (mm/hr)	23.6 (21.1)	31.3 (25.3)	25.8 (22.6)
CRP (mg/dL)	1.4 (2.6)	2.0 (3.5)	1.6 (2.9)
RA medication use, n (%)			
Prednisone	952 (47)	373 (53)	1,325 (47)
csDMARDs	1,341 (67)	514 (66)	1,325 (47)
b/tsDMARDs	602 (30)	197 (25)	799 (29)

* % is percentage of nonmissing. Missing data: n = 321 education, n = 86 body mass index, n = 70 smoking status, n = 62 RA disease duration, n = 91 DAS28, n = 23 tender joint count, n = 23 swollen joint count, n = 326 patient global, n = 1,050 provider global, n = 83 CDAI, n = 60 ESR, n = 121 CRP, n = 96 anti‐CCP antibody. b/tsDMARD, biologic and targeted synthetic disease‐modifying antirheumatic drug; CCP, cyclic citrullinated peptide; CDAI, Clinical Disease Activity Index; CRP, C‐reactive protein; csDMARD, conventional synthetic disease‐modifying antirheumatic drug; DAS28, Disease Activity Score in 28 joints; ESR, erythrocyte sedimentation rate; MDHAQ, Multidimensional Health Assessment Questionnaire; RA, rheumatoid arthritis; VA, Veterans Affairs.

^a^ American Indian or Alaska Native, Asian, multirace, Native Hawaiian or Pacific Islander.

### Cross‐sectional analyses

#### Association between RA disease activity and frailty at enrollment

Higher RA disease activity was positively associated with higher VAFI score at baseline after covariate adjustment (adjusted β = 0.86, 95% confidence interval [CI] 0.63–1.08, *P* ≤ 0.001).

#### Secondary analyses

Among the individual components of RA disease activity, PtGA, PrGA, TJC, ESR, and CRP were all significantly and positively associated with higher VAFI scores at VARA registry enrollment, but SJC was not (Table 2).

**Table 2:** Unadjusted and adjusted regression models evaluating the association of RA disease activity and its components with VA Frailty Index score at baseline (N = 2,800)*

	β^a^	95% CI	*P* value	adj β^b^	95% CI	*P* value
RA disease activity measures						
DAS28 score	0.71	0.47–0.95	<0.001	0.86	0.63–1.08	<0.001
DAS28 category^c^						
Remission	–			–		
Low	1.94	0.74–3.13	0.002	1.93	0.81–3.04	0.001
Moderate	2.49	1.54–3.44	<0.001	2.67	1.78–3.55	<0.001
High	3.15	2.00–4.30	<0.001	3.88	2.81–4.95	<0.001
CDAI score	0.04	0.01–0.07	0.013	0.07	0.04–0.10	<0.001
CDAI category						
Remission	–			–		
Low	0.91	−0.37 to 2.19	0.164	0.97	−0.22 to 2.16	0.110
Moderate	1.71	0.42–2.99	0.009	2.29	1.07–3.51	<0.001
High	1.69	0.32–3.05	0.015	2.74	1.47–4.00	<0.001
Components of RA disease activity^d^						
PtGA (0–100)	0.46	0.31–0.61	<0.001	0.58	0.44–0.72	<0.001
PrGA (0–100)	0.19	0.01–0.36	0.035	0.31	0.15–0.48	<0.001
TJC (0–28)	0.01	−0.05 to 0.07	0.707	0.09	0.03–0.14	0.001
SJC (0–28)	−0.06	−0.13 to 0.01	0.090	−0.01	−0.08 to 0.06	0.755
ESR, mm/dL	0.78	0.62–0.09	<0.001	0.65	0.50–0.81	<0.001
CRP, mg/dL	0.29	0.17–0.04	<0.001	0.28	0.16–0.39	<0.001

* RA disease activity composite scores, categories, and components evaluated in separate models. CDAI, Clinical Disease Activity Index; CI, confidence interval; DAS28, Disease Activity Score in 28 joints; ESR, erythrocyte sedimentation rate; PrGA, provider global assessment; PtGA, patient global assessment; RA, rheumatoid arthritis; SJC, swollen joint count; TJC, tender joint count; VA, Veterans Affairs.

^a^ Values are beta coefficients ×100 for predicting VA Frailty Index.

^b^ Models adjusted for baseline age, sex, race, education, body mass index, smoking status, RA disease duration, and anti–cyclic citrullinated peptide antibody positivity.

^c^ Remission was the referent for DAS28 and CDAI categories.

^d^ Coefficients for PtGA, PrGA, and ESR pertain to a 10‐unit increase for predicting VA Frailty Index.

#### Sensitivity analyses

When the CDAI composite score was substituted for the DAS28 in the original model, the relationship between RA disease activity and frailty was attenuated but remained statistically significant even after controlling for age, sex, race, education, BMI, smoking status, and RA disease duration (adjusted β = 0.07, 95% CI 0.04–0.10, *P* < .001; Table 2). Compared to remission, patients with moderate (adjusted β = 2.29, 95% CI 1.07–3.51) and high (adjusted β = 2.74, 95% CI 1.47–4.00) disease activity by CDAI had significantly higher frailty scores (*P* ≤ 0.05). Point estimates predicting VAFI score did not substantially change when RA medication use (prednisone, csDMARD, b/tsDMARD) was included in the original model (data not shown).

### Longitudinal analyses

There were 2,786 veterans included in the longitudinal analyses who had one or more DAS28 scores following enrollment, and they averaged 4.1 visits over five years (range 1–5). The percentage of frail participants increased slightly from 40% (1,102 of 2,768) to 45% (767 of 1,713) over five years. The mean VAFI score of VARA participants increased incrementally over five years: 0.201 ± SD 0.111 (n = 2,786) in year 1, 0.205 ± SD 0.116 (n = 2,555) in year 2, 0.208 ± SD 0.118 (n = 2,289) in year 3, 0.210 ± SD 0.122 (n = 2,007) in year 4, and 0.218 ± SD 0.124 (n = 1,713) in year 5 (Figure 3). A continuous plot, distribution of the mean VAFI values, and a comparison of mean VAFI values in those who died and exited the study are shown in Supplement 2.

**Figure 3:**
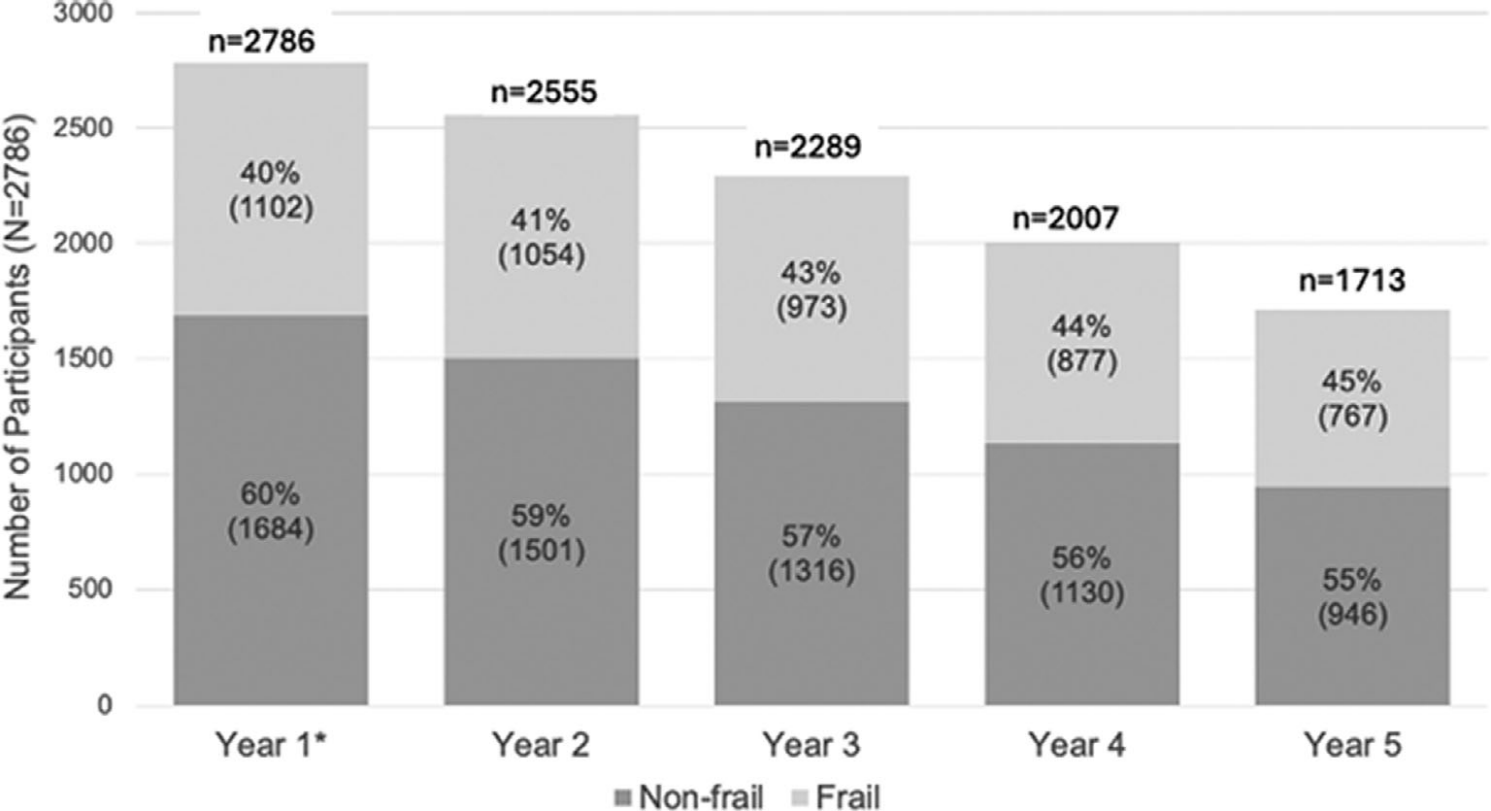
The proportion of frail and non‐frail participants over 5 years of observation. *Year 1 marks the end of the 3 years after enrollment in the VA Rheumatoid Arthritis registry, allowing for the 3‐year lag between DAS28 and VA Frailty Index calculation, which is derived from diagnostic codes accrued over the prior 3 years. DAS28, Disease Activity Score in 28 joints; VA, Veterans Affairs.

#### RA disease activity and frailty over time

In the longitudinal analyses, higher RA disease activity was associated with higher VAFI scores when controlling for potential confounders (adjusted β 0.53, 95% CI 0.26–0.80, *P* < 0.001; Table 3). A very small but significant negative interaction between RA disease activity and follow‐up duration (year) was maintained in the adjusted model (adjusted β −0.09, 95% CI −0.19 to 0.01, *P* = 0.073, prespecified α = 0.20), suggesting the relationship between disease activity and frailty may slightly attenuate each year. Given the small magnitude of the interaction (the coefficient was more than five times the interaction term) and the CI included zero, this finding was exploratory. Missing data are shown in the Table 3 footnote. Missingness of baseline covariates was low (≤5% for all variables) except education level (11%). Missingness of mean DAS28 years 1 to 5 was ≤16%: year 1 (15%), year 2 (4%), year 3 (13%), year 4 (15%), year 5 (16%). Complete case models were compared to the imputed models, and the SEs from the imputed models were consistently smaller, so missing data were unlikely to influence study findings.^25^, ^29^

**Table 3:** Unadjusted and adjusted regression generalized estimation equation population‐average models evaluating the relationship of rheumatoid arthritis disease activity with frailty over up to 5‐year (n = 2,786)*

	β^a^	95% CI	adj β^b^	95% CI
DAS28 score (3‐y lookback)^c^	0.49	0.26–0.73	0.53	0.26–0.80
DAS28 score (1‐y lookback)	0.44	0.29–0.58	0.49	0.35–0.64

* Missing data: n = 321 education, n = 86 body mass index, n = 70 smoking status, n = 62 RA disease duration, n = 96 anti‐CCP antibody, n = 444 mean DAS28–year 1, n = 116 mean DAS28–year 2, n = 319 mean DAS28–year 3, n = 319 mean DAS28–year 4, n = 283 mean DAS28–year 5. anti‐CCP, anti–cyclic citrullinated peptide; DAS28, Disease Activity Score in 28 joints; CI, confidence interval; VAFI, Veterans Affairs Frailty Index.

^a^ Values are beta coefficients for VAFI ×100 (all *P* < 0.001).

^b^ Model adjusted for baseline age, sex, race, education level, body mass index, smoking status, rheumatoid arthritis disease duration, and anti‐CCP antibody positivity.

^c^ Only models evaluating DAS28 score and VAFI (3‐year lookback) included the interaction term (DAS28–year number).

#### Sensitivity analysis

We explored an alternative length of assessment period for the VAFI in which the VAFI was calculated using a one‐year lookback and one‐year time lag after the DAS28 score. Point estimates shown in Table 3 were similar to the original adjusted model (adjusted β 0.49, 95% CI 0.35–0.64, *P* < 0.001). However, the interaction between RA disease activity and follow‐up duration (year) was not significant, and thus not included.

## DISCUSSION

Using a population level approach, we demonstrated that higher baseline RA disease activity was associated with higher VAFI scores over five years of follow‐up independent of important covariates. We also observed independent relationships between several components of RA disease activity (PtGA, PrGA, TJC, ESR, CRP) and frailty at baseline to offer additional insight and help disentangle the relationship between these two dynamic concepts.

Our findings suggest that people with higher RA disease activity were more likely to experience greater frailty burden over time, which may make them more susceptible to poor long‐term outcomes such as hospitalization, disability, and death.^4^, ^7^, ^12^ Lowering RA disease activity in frail people may be beneficial to improve outcomes. In the context of existing literature, early improvement of frailty scores has been observed relative to lower disease activity in patients with newly diagnosed RA,^7^ whereas we observed that higher RA disease activity was linked with a sustained increase in the VAFI scores of veterans over five years without early improvement, likely due to our cohort's longer RA disease duration and lower baseline disease activity. Although the association between RA disease activity and frailty persisted, a small negative interaction between RA disease activity and follow‐up duration suggests the predictive value of high disease activity may decrease over time, perhaps as aging‐related frailty becomes a greater contributor.

We observed a similar relationship between disease activity and frailty without attenuation in the relationship when using a more temporally precise VAFI with a shorter period of health deficit accumulation supporting the possibility of a more direct and sustained relationship between current RA disease activity and frailty. The longitudinal associations observed in our study highlight the importance of ongoing RA disease control to minimize frailty, particularly in people with established RA disease. Achieving a target of low disease activity or remission in frail people is not without challenges because they are likely to be older with increasing morbidities and polypharmacy and susceptible to infection.^30^, ^31^ Future studies are needed to identify specific thresholds of RA disease activity that could benefit frail adults.

Although a minimally clinically important difference has not been established for the VAFI, a 0.10‐point increase is commonly viewed as meaningful because it corresponds to transitions across validated frailty categories (≤0.10 nonfrail; >0.10–0.20 prefrail; >0.20–0.30 mildly frail; >0.30–0.40 moderately frail; >0.40 severely frail).^20^, ^21^, ^23^, ^32^ In our study, patients with high disease activity (by DAS28) had VAFI scores that were on average 0.0388 points higher than those in remission after adjustment for sociodemographic and clinical factors. Because the VAFI is constructed from 31 deficits, this magnitude approximates the accumulation of one additional health deficit, representing a modest absolute increase that reflects a statistically significant and independent association between disease activity and frailty burden, supporting its relevance as a contributor to vulnerability in RA.

We found that disease activity components related to systemic inflammation and perceived RA disease burden had a cross‐sectional association with frailty, although localized joint pathology, or the SCJ, did not. These findings suggest that inflammation and the subjective impact of RA disease may contribute to frailty development and progression more than localized joint pathology alone.^33^, ^34^ Mitigating frailty in the setting of active RA disease thus calls for management strategies beyond RA‐specific treatments to address latent factors such as pain, depression, and multimorbidity that underly global assessments of disease activity in RA.^6^, ^35^, ^36^ This is particularly relevant for veterans, who often face chronic pain, posttraumatic stress disorder, and higher rates of comorbid conditions.^37^, ^38^ Elevations in ESR and CRP represent systemic inflammation, which often accompanies or exacerbates sarcopenia, a disproportionate loss of muscle mass or strength and a known hallmark of frailty.^39^, ^40^ Incorporating structured exercise and nutrition goals aimed at preserving strength and function may further support reductions in frailty and enhance resilience in RA.^41^, ^42^ Future research should explore the impact of multidisciplinary care on frailty in RA, veterans’ experiences, and protective factors like resilience to inform personalized interventions and promote healthy aging in RA populations.

We demonstrated frailty in RA is not limited to older adults, with a 28% frailty prevalence in our cohort (mean age 64 years) at VARA registry enrollment, and over half of frail veterans were younger than 60 years. Comparable frailty proportions have been documented, including a pooled prevalence of 34% among RA cohorts aged 51 to 74 years.^1^ Our findings reinforce the need for early assessment in younger adults with chronic conditions.^6^, ^43^, ^44^, ^45^ Identifying frailty early may help address secondary frailty, which arises from conditions beyond traditional age‐related frailty like low bone mineral density precipitating an increased risk for falls and fracture.^5^, ^46^, ^47^, ^48^ Targeted interventions including vitamin D optimization, deprescribing, weight‐bearing exercise, and smoking cessation could help mitigate secondary frailty and promote healthy aging. Further studies should investigate early frailty screening in RA and explore ways to prevent or reduce secondary frailty and its related health risks.

A strength of this study was the ability to assess frailty longitudinally among a large national cohort of real‐life patients with RA. This was the first known study to examine RA disease activity and frailty in adults with established RA over five years of follow‐up using a population‐average approach. With a large sample size and access to several predictors, we controlled for a wide range of factors known to confound the relationship between RA disease activity and frailty. Male patients composed 89% of our study population representing an understudied demographic in RA research.^49^ This study had limitations. Disproportionately fewer women may limit external validity or generalization to other RA populations. Veterans could have received care outside the VA system, which may have resulted in omitted codes, VAFI underestimation, or misclassification of frailty. We did not evaluate for death, and there may have been a disproportionate number of frail individuals who died and exited the study in the longitudinal analysis. Validation of the VAFI with other frailty measures in RA populations is needed.

Disease activity has both a cross‐sectional and longitudinal relationship with frailty in RA. Improvements in RA disease activity control may aid in delaying frailty onset or progression. Future studies should evaluate whether achieving remission or low RA disease activity and providing multidisciplinary interventions aimed at pain control, mental health, and optimizing care of other chronic conditions in veterans with RA improve frailty status.

## AUTHOR CONTRIBUTIONS

All authors contributed to at least one of the following manuscript preparation roles: conceptualization AND/OR methodology, software, investigation, formal analysis, data curation, visualization, and validation AND drafting or reviewing/editing the final draft. As corresponding author, Dr Loecker confirms that all authors have provided the final approval of the version to be published and takes responsibility for the affirmations regarding article submission (eg, not under consideration by another journal), the integrity of the data presented, and the statements regarding compliance with institutional review board/Declaration of Helsinki requirements.

## Supporting information


**Disclosure Form**:

**Supplement 1**:

**Supplement 2**:
