# Supplementary material for: Associations of Rheumatoid Arthritis Disease Activity With Frailty Over Five Years of Follow‐up
Source: Arthritis Care Res (Hoboken). 2026 May 5;78(11):1759–68. doi: 10.1002/acr.80056 (PMC13710778; doi:10.1002/acr.80056)
Supplement: Supplementary file 2 [file ACR-78-1759-s001.docx]

# Veterans Affairs Frailty Index Components and Accompanying Codes (Orkaby, et al. 2019)

| **VA-Frailty Index Variable** | **ICD-9 Code** | **ICD-9 Procedure Code** | **CPT Code** | **HCPCS Code** |
| --- | --- | --- | --- | --- |
| **Morbidity:** | | | | |
| 1-Anemia | 280.0-285.0 |  |  |  |
| 2-Atrial Fibrillation | 427.31 | 37.34 | 93656-93657, 93799 | 93656-93657, 93799 |
| 3-Cancer (any except basal cell skin cancer) | 140-160, 170.0-172.0  174.0-179.0, 180-200 |  |  |  |
| 4-Cerebrovascular disease: Stroke/TIA | 362.34  433.0-435.0, 436.0, 437.0-437.1  438.0-438.5,  438.81-438.82,  438.89, 438.9 |  |  |  |
| 5-Coronary Artery Disease: MI/CABG/PCI | 410-412  414  429.2  429.5  429.7  V45.82 | 00.66, 0.66,  36.01-36.02, 36.05,  36.10-36.17, 36.19 | 33510-33514, 33516-33523, 33533-33536, 33572, 92920-92921, 92924-92925,92933-92934, 92937-92938, 92941, 92943-92944, 92982, 92984, 92995-92996, 93540, 93564, 93570, C9602-C9608, S2205-S2209 | 33510-33514, 33516-33523, 33533-33536, 33572, 92920-92921, 92924-92925,92933-92934, 92937-92938, 92941, 92943-92944, 92982, 92984, 92995-92996, 93540, 93564, 93570, C9602-C9608, S2205-S2209 |
| 6-Diabetes | 250.00, 250.02, 250.10, 250.12, 250.20, 250.22, 250.30, 250.32, 250.40, 250.42, 250.50, 250.52, 250.60, 250.62, 250.70, 250.72, 250.80, 250.82, 250.90, 250.92 |  |  |  |
| 7-Heart Failure (diastolic or systolic) | 428, 402.01, 402.11, 402.91, 404.01, 404.03, 404.11, 404.13, 404.91, 404.93 |  |  |  |
| 8-Hypertension | 401-405 |  |  |  |
| 9-Kidney Disease  (Chronic Kidney Disease code = 0.5,  Dialysis code =1) | Chronic Kidney Disease:  250.4, 403.00, 403.10, 403.90, 404.00, 404.01, 404.10-404.11, 404.90-404.91, 582, 585-588    Dialysis:  403.01, 403.11, 403.91, 404.02-404.03, 404.12-404.13, 404.92-404.93, V42.0, V56.0, V45.1, E879.1 | 39.93, 39.95, 54.98 | 0505F, 0507F, 36800, 36810, 36815, 4052F-4055F, 90921, 90925, 90935, 90937, 90939-90940, 90960-90962, 90966, 90970, 90976-90977, 90982-90983, 90988-90995, 90998-90999, 93990, 99512, 99559, E1590, E1699, G0257, G0323, G0327, G8075-G8076, G8081-G8082, G8085 | 0505F, 0507F, 36800, 36810, 36815, 4052F-4055F, 90921, 90925, 90935, 90937, 90939-90940, 90960-90962, 90966, 90970, 90976-90977, 90982-90983, 90988-90995, 90998-90999, 93990, 99512, 99559, E1590, E1699, G0257, G0323, G0327, G8075-G8076, G8081-G8082, G8085 |
| 10-Liver Disease or Cirrhosis | 531-534, 570-571, 572.2, 572.3, 572.8, 573 |  |  |  |
| 11-Lung disease: COPD or Asthma | 490-496, 510 |  |  |  |
| 12-Thyroid Disease | 242, 244-245, 246.0, 246.3-246.9 |  |  |  |
| 13-Osteoporosis or osteoporosis related fracture (vertebral fractures) | 733.00, 733.13 |  |  |  |
| 14-Incontinence | 625.6, 787.6, 788.3, 788.91 |  |  |  |
| **Function:** | | | | |
| 15-Arthritis (~~rheumatoid arthritis~~ or osteoarthritis) | 274, 446.5, 710.9,  714.0-714.2, 714.4, 714.89, 714.9, 715,  716.1-716.3,  716.5-716.6, 716.8-716.9, 725 |  |  |  |
| 16-Use of Durable Medical Equipment |  |  | E0000, E0100, E0105, E0130, E0135, E0140, E0153-E0159, E0160, E0170-E0171, E0240, E0245, E0247-E0248, E0291, E0293, E0295, E0297, E0303-E0304, E0621, E0625, E0630, E0637-E0638, E0641-E0642, E0950, E0961, E0966-E0967, E0790, E0972-E0979, E0981-E0986, E0988, E0990-E0993, E0995, E1002- E1012, E1014-E1018, E1028-E1031, E1035-E1036, E1038-E1039, E1065-E1066, E1069, E1160-E1161, E1220-E1228, E1230-E1238, E1296-E1298, E2200, E2210-E2215, E2218- E2222, E2224-E2228, E2231, E2291, E2293-E2294, E2300-E2301, E2310-E2311, E2320, E2330-E2331, E2340-E2343, E2350-E2351, E2358-E2359, E2360, E2370-E2378, E2380, E2390-E2397, E2600, E2610, E2620, E2630-E2633, K0000, K0020, K0030, K0040, K0080, K0050- K0058, K0062-K0063, K0069-K0073, K0077, K0079, K0080, K0098, K0103, K0105, K0107-K0108, K0195, K0460-K0461, K0650, K0660, K0800-K0802, K0806-K0808, K0812-K0816, K0820, K0830-K0831, K0835-K0843, K0848-K0849, K0850, K0860-K0864, K0868-K0871, K0877-K0880, K0884-K0886, K0898-K0900, T1000-T1005, T1019-T1022, T1030-T1031 | E0000, E0100, E0105, E0130, E0135, E0140, E0153-E0159, E0160, E0170-E0171, E0240, E0245, E0247-E0248, E0291, E0293, E0295, E0297, E0303-E0304, E0621, E0625, E0630, E0637-E0638, E0641-E0642, E0950, E0961, E0966-E0967, E0790, E0972-E0979, E0981-E0986, E0988, E0990-E0993, E0995, E1002- E1012, E1014-E1018, E1028-E1031, E1035-E1036, E1038-E1039, E1065-E1066, E1069, E1160-E1161, E1220-E1228, E1230- E1238, E1296-E1298, E2200, E2210-E2215, E2218- E2222, E2224-E2228, E2231, E2291, E2293-E2294, E2300-E2301, E2310-E2311, E2320, E2330-E2331, E2340-E2343, E2350-E2351, E2358-E2359, E2360, E2370-E2378, E2380, E2390-E2397, E2600, E2610, E2620, E2630-E2633, K0000, K0020, K0030, K0040, K0080, K0050- K0058, K0062-K0063, K0069-K0073, K0077, K0079, K0080, K0098, K0103, K0105, K0107-K0108, K0195, K0460-K0461, K0650, K0660, K0800-K0802, K0806-K0808, K0812-K0816, K0820, K0830-K0831, K0835-K0843, K0848-K0849, K0850, K0860-K0864, K0868-K0871, K0877-K0880, K0884-K0886, K0898-K0900, T1000-T1005, T1019-T1022, T1030-T1031 |
| 17-Fall or Fall-related diagnoses:  -hip fractures  -subdural hematoma  -subarachnoid hematoma | 4300, 733.14, 733.96, 8000-8010, 8030, 8350, 8520,  E8800,  E884.2-E884.9, E885.9, E8870, E8880 |  |  |  |
| 18-Fatigue | 780.71, 780.79 |  |  |  |
| 19-Gait Abnormality or difficulty walking | 719.70, 781.20-781.40 |  |  |  |
| 20-Parkinson’s Disease and Tremors | 3320, 333.10 |  |  |  |
| 21-Peripheral vascular disease or Intermittent claudication | 4400-4440, 4470,  4510-4530, 5570 |  |  |  |
| 22-Muscular wasting and disuse atrophy/ Cachexia/ Debility | 307.10, 728.20, 728.87, 783.00, 799.30-799.40 |  |  |  |
| **Sensory Loss:** | | | | |
| 23-Hearing Impairment/  Hearing Aid | 388.00-388.20 |  | 92590-92596, S0618, V5010-V5011, V5014, V5030, V5040, V5050, V5060, V5070, V5080, V5095, V5100,  V5110, V5130,  V5140, V5150,  V5170, V5180,  V5190, V5210,  V5220, V5230, V5240, V5250,  V5260-V5263 | 92590-92596, S0618, V5010-V5011, V5014, V5030, V5040, V5050, V5060, V5070, V5080, V5095, V5100,  V5110, V5130,  V5140, V5150,  V5170, V5180,  V5190, V5210,  V5220, V5230,  V5240, V5250,  V5260-V5263 |
| 24-Peripheral Neuropathy | 250.70, 337.00, 337.09  337.10, 356.40, 356.80, 357.10-357.70 |  |  |  |
| 25-Vision Comorbidity (macular degeneration, glaucoma, blindness) | 362.50-362.53,  365.05-365.13,  365.20-365.70, 365.81-365.82,  365.89, 365.90,  368.30-368.31,  368.40, 368.60,  368.62-368.69, 368.7, 368.80-368.90, 377.75 |  |  |  |
| **Cognition and Mood:** | | | | |
| 26-Dementias (Alzheimer’s, Vascular, Lewy Body, Pick’s disease, Mild Cognitive Impairment, etc.) | 290.00-290.40,  291.10-291.20,  293.00-293.10,  294.80-294.90, 331.00, 331.10-331.11, 331.82-331.83, 331.92, 333.40, 438.00, 780.09, 780.93, 799.50 |  |  |  |
| 27-Anxiety | 293.84,  300.00-300.10, 309.21, 309.24, 309.28 |  |  |  |
| 28-Depression/Bipolar | 2960, 298.00,  309.00-309.10, 3110 |  |  |  |
| **Other:** | | | | |
| 29-Chronic Pain | 338.29, 724.50 |  |  |  |
| 30-Failure to thrive | 783.70 |  |  |  |
| 31-Weight loss in the past year | 783.20 |  |  |  |
