# Supplementary material for: Associations of Rheumatoid Arthritis Disease Activity With Frailty Over Five Years of Follow‐up
Source: Arthritis Care Res (Hoboken). 2026 May 5;78(11):1759–68. doi: 10.1002/acr.80056 (PMC13710778; doi:10.1002/acr.80056)
Supplement: Supplementary file 3 [file ACR-78-1759-s003.docx]

Plot of Continuous Mean VA Frailty Index Values by Year

VA Frailty Index

| Year | VA Frailty Index (3-yr lookback) | VA Frailty Index (1-yr lookback) |
| --- | --- | --- |
| 1 |  | 0.139 |
| 2 |  | 0.139 |
| 3 | 0.201 | 0.144 |
| 4 | 0.205 | 0.148 |
| 5 | 0.208 | 0.151 |
| 6 | 0.21 |  |
| 7 | 0.218 |  |

Distribution of VA Frailty Index by Year

| Year(s) after VARA enrollment | Mean VA Frailty Index  3-year lookback  (mean, SD, n) | Mean VA Frailty Index  1-year lookback  (mean, SD, n) |
| --- | --- | --- |
| 1 | _ | 0.139±0.082 (n=2,783) |
| 2 | _ | 0.139±0.083 (n=2,518) |
| 3 | 0.201±0.11 (n=2,786) | 0.144±0.087 (n=2,227) |
| 4 | 0.205±0.12 (n=2,555) | 0.147±0.089 (n=1975) |
| 5 | 0.208±0.12 (n=2,289) | 0.151±0.091 (n=1,680) |
| 6 | 0.210±0.12 (n=2,007) | _ |
| 7 | 0.218±0.12 (n=1,713) | _ |

A comparison of VA Frailty Index values and frailty status at VA Rheumatoid Arthritis registry enrollment between those who survived and died by outcome year (N=2800)

| VA Frailty Index Outcome Year | Status | N (%) | Mean VA Frailty Index** | Frail (VAFI ≥0.20)  N (%) |
| --- | --- | --- | --- | --- |
| Year 1* | Survived | 2678 (96%) | 0.166±0.098 | 733 (27%) |
|  | Died | 122 (4%) | 0.199±0.089 | 52 (43%) |
| Year 2 | Survived | 2699 (96%) | 0.166±0.097 | 744 (27%) |
|  | Died | 101 (4%) | 0.202±0.109 | 41 (41%) |
| Year 3 | Survived | 2705 (97%) | 0.167±0.097 | 747 (28%) |
|  | Died | 95 (3%) | 0.195±0.105 | 38 (40%) |
| Year 4 | Survived | 2720 (97%) | 0.167±0.097 | 756 (28%) |
|  | Died | 80 (3%) | 0.194±0.103 | 29 (36%) |
| Year 5 | Survived | 2717 (97%) | 0.167±0.098 | 765 (28%) |
|  | Died | 83 (3%) | 0.174±0.097 | 20 (24%) |

*Year 1 refers to 3 years after VA Rheumatoid Arthritis registry enrollment.

**VA Frailty Index (VAFI) calculated at registry enrollment
